# (Nitrito-κ*O*)(nitro-κ*N*)(nitrosyl-κ*N*)bis­(tri­phenylphosphane-κ*P*)rhodium(III)

**DOI:** 10.1107/S241431462500598X

**Published:** 2025-07-08

**Authors:** Daniel R. Albert, Michael Gau, Edward Rajaseelan

**Affiliations:** a Department of Chemistry Millersville University,Millersville PA 17551 USA; b Department of Chemistry, University of Pennsylvania, Philadelphia, PA 19104, USA; Benemérita Universidad Autónoma de Puebla, México

**Keywords:** crystal structure, rhodium, nitrito, nitros­yl, nitro

## Abstract

The structure of the title compound, [Rh(NO)(NO_2_)_2_(C_18_H_15_P)_2_] or [Rh(NO)(NO_2_)(ONO)(PPh_3_)_2_], has been determined by single-crystal X-ray diffraction. A previous report of the title compound showed it crystallized in a monoclinic space group [Rajaseelan *et al.* (1999 ▸). *J. PA. Acad. Sci.***73**, 63–66; refcode SASTOW in the CSD]. However, it was unable to be refined because of severe disorder. In this study, two distinct crystals of the title compound were found showing the compound to be polymorphic. One of the crystals was unable to be resolved but was found to be in a monoclinic space group, as in the previously reported study. The other crystal was able to be refined and crystallizes in the triclinic space group *P*1. The refined structure consists of two discrete monomeric mol­ecules per unit cell. The mol­ecular geometry around rhodium is that of a distorted square pyramid, with nitro­gen of the nitro ligand, oxygen of the nitrito ligand and phospho­rus atoms of the phosphane ligands lying in the basal plane and the nitro­gen atom of the bent nitrosyl occupying the apical position. The nitrosyl ligand exhibits positional disorder whereas the nitro and nitrito ligands show disorder across coordination sites with the disorder modelled in a 0.91:0.09 ratio. Both intra­molecular C—H ⋯O (nitro and nitrito) and inter­molecular C—H ⋯N (nitrito) inter­actions are observed. There are no hydrogen-bonding inter­actions with the N or O atoms of the nitrosyl ligand.

## Abstract 2

The title Rh^III^ complex is a rare example of a mononuclear compound gathering nitrito, nitro and nitrosyl ligands.

## Graphical abstract

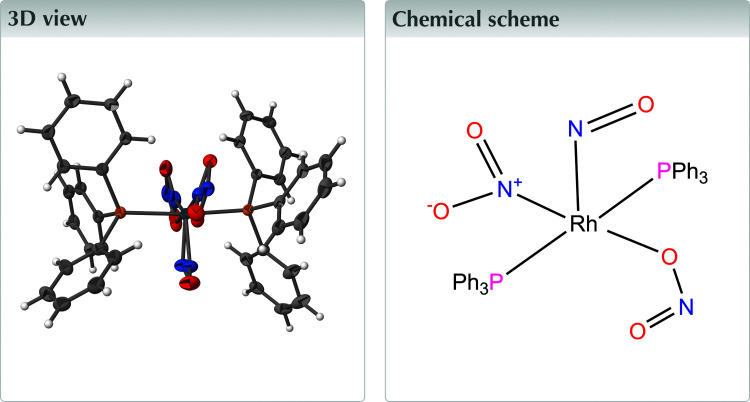

## Structure description

Nitrosyl is a versatile ligand and synthetic, structural, chemical reactivity, and spectroscopic studies of transition metal nitrosyl complexes are of inter­est due to the role of NO in biochemical systems and in coordination chemistry (Machura, 2005 ▸; Daniel & Gourlaouen, 2019 ▸). Nitrite coordinates to transition metals in different bonding modes. The different *M*NO_2_ isomers are characterized by distinct infrared bands, ^15^N-NMR chemical shifts, and crystal structures of these linkages (Feltham, 1989 ▸). Synthesis and structures of many rhodium nitrosyl and nitrite complexes have been reported (English *et al.*, 1987 ▸; Cheung *et al.*, 2007 ▸; Gaviglio *et al.*, 2009 ▸; Singh *et al.*, 2011 ▸; Vorobyeva *et al.*, 2022 ▸). The title compound has previously been reported and characterized with the bonding modes of the NO_*x*_ groups confirmed by the infrared spectra, ^15^N labelling studies, and multinuclear NMR spectra (Rajaseelan *et al.*, 1999 ▸).

**Figure 1:**
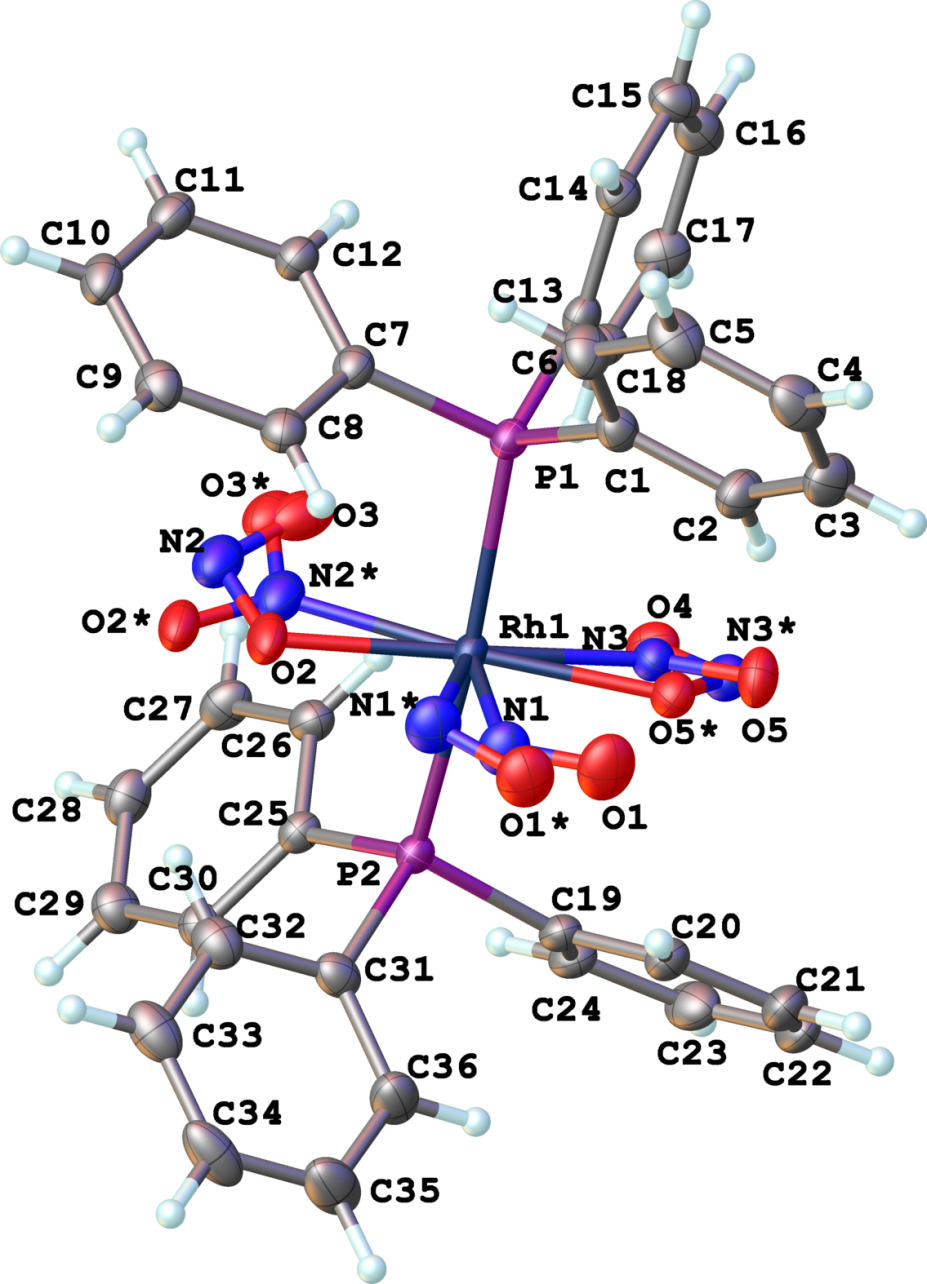
Mol­ecular structure of the title compound shown with displacement ellipsoids at the 50% probability level.

**Figure 2:**
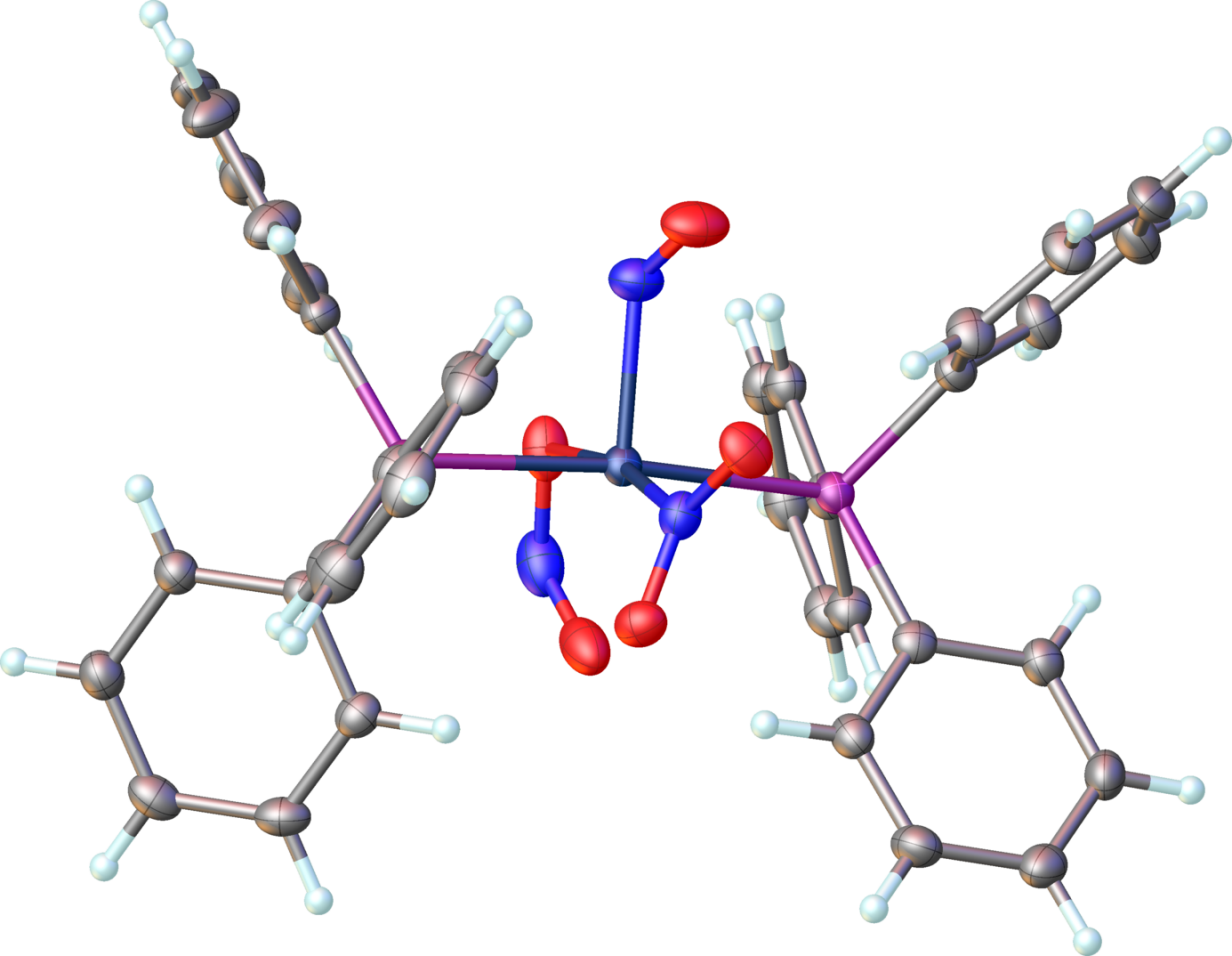
A perspective view of the main component (91%) title compound displaying the distorted square-pyramidal coordination around Rh1 with the nitrosyl ligand in the apical position.

The title complex, [Rh(NO)(NO_2_)(ONO)(PPh_3_)_2_], consists of two well-separated monomeric structural units per unit cell. There is no crystallographically imposed twofold axis on the mol­ecule and the NO_*x*_ ligands are disordered and refined with a ratio of 0.91:0.09 as shown in Fig. 1 ▸. The coordination sphere around rhodium, formed by nitrosyl, nitro, nitrito, and two tri­phenyl­phosphane ligands, results in a distorted square-pyramidal environment as shown in Fig. 2 ▸. The square pyramidal shape is supported by τ_5_ parameters (Addison *et al.*, 1984 ▸) of both the main (τ_5_ = 0.078) and disordered (τ_5_ = 0.062) structures that are close to zero. The nitro­gen atom of the nitrosyl ligand which coordinates in a bent fashion, occupies the apical position. The Rh1—N1 [Rh1—N1*] distance is 1.938 (3) [1.93 (3)] Å and the Rh1—N1—O1 [Rh1—N1*—O1*] bond angle is 126.1 (2) [111 (2)]°. The two phosphane ligands occupy the sterically favoured *trans* positions in the basal plane. The Rh1—P1 and Rh1—P2 bond lengths are 2.4001 (6) and 2.3978 (6) Å, respectively, and the P2—Rh1—P1 bond angle is 173.609 (19)°. The nitrito (–ONO) ligand is in the *endo*-conformation with a Rh1—O2 [Rh1—O5*] bond length of 2.1105 (19) [2.09 (2)] Å, and a O2—N2—O3 [O5*—N3*—O4] bond angle of 114.1 (3) [116 (2)]°. The nitro­gen atom of the nitro (–NO_2_) group occupies the other position on the basal plane and is *trans* to the oxygen of the nitrito (–ONO) ligand. The Rh1—N3 [Rh1—N2*] bond length is 2.019 (2) [2.03 (3)] Å and the O5—N3—O4 [O2*—N2*—O3*] bond angle is 120.3 (2) [118 (3)]°, as expected for an *sp*^2^ hybridized nitro­gen atom.

**Figure 3:**
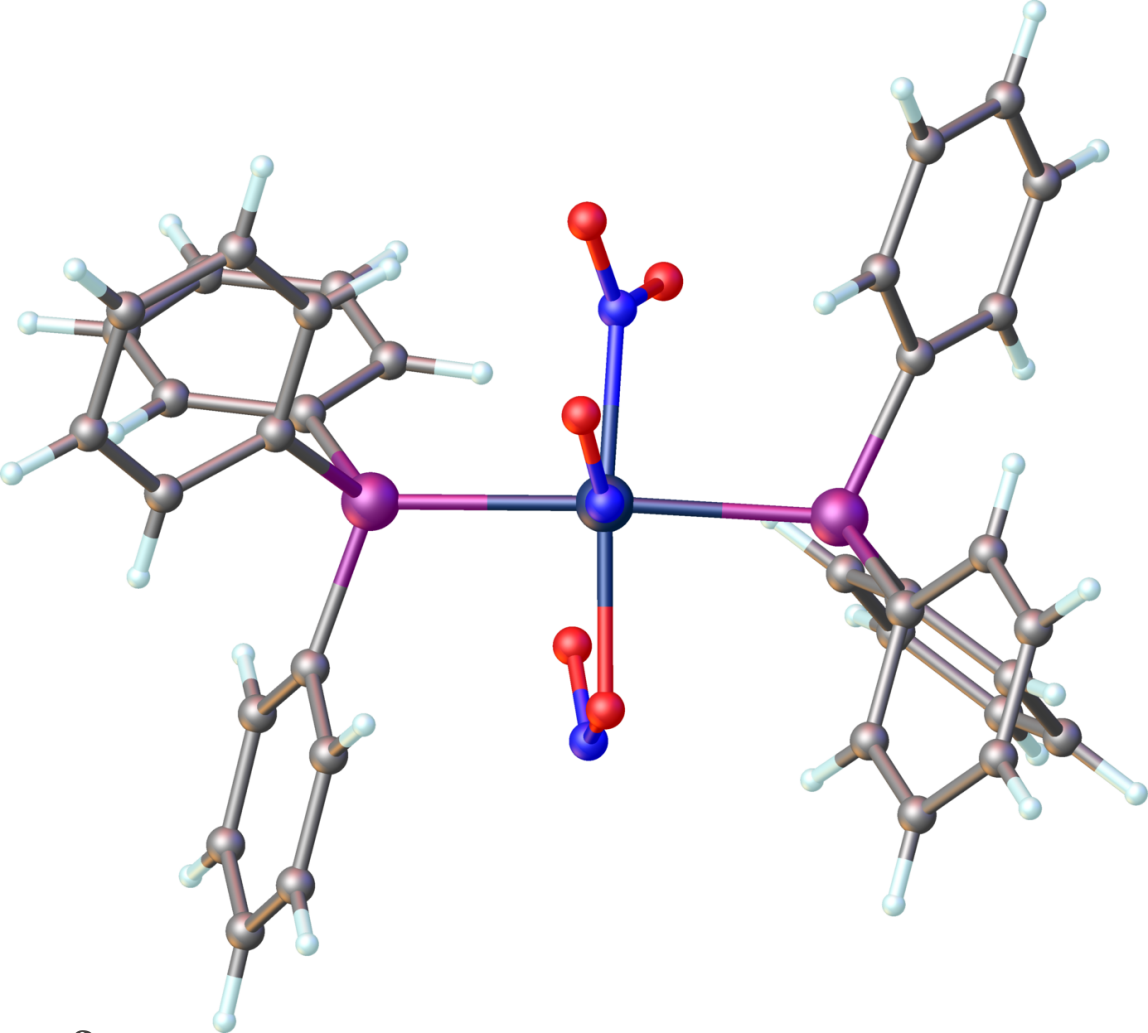
A perspective view of the main component (91%) of the title compound showing the bent nitrosyl ligand oriented along the N—Rh—O plane.

**Figure 4:**
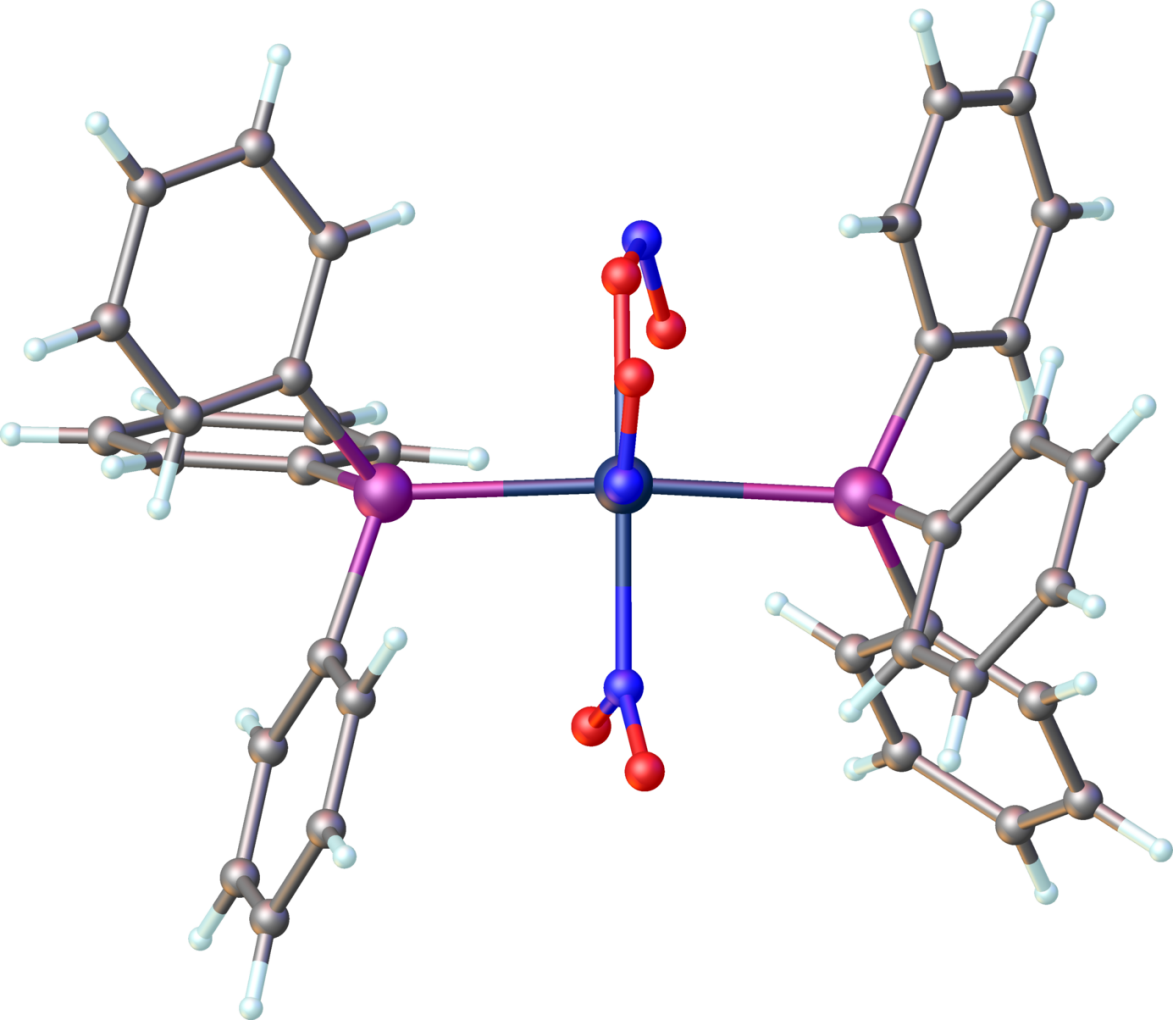
A perspective view of the minor disordered component (9%) of the title compound showing the bent nitrosyl ligand oriented along the N—Rh—O plane.

It has been proposed and supported by mol­ecular orbital calculations that the nitrosyl group bends in the direction of the strongest π-acceptor ligand coordinating in the basal plane (Hoffmann *et al.*, 1974 ▸; Ibers & Mingos, 1971 ▸; Pierpont & Eisenberg, 1972 ▸). In Rh(NO)Cl_2_(PPh_3_)_2_ the nitrosyl ligand lies in the P—Rh—P plane (Goldberg *et al.*, 1975 ▸). The NO_2_^−^ ligand is a better π-acceptor than PPh_3_ (Comas-Vilà & Salvador, 2024 ▸) and in the title complex the nitrosyl ligand lies approximately in the O(nitrito)—Rh—N(nitro) plane. This is clearly indicated in Fig. 3 ▸ (major component) and Fig. 4 ▸ (minor disordered component). The dihedral angle formed by the Rh1/N1/O1 and the O2/Rh1/N3 planes is 25.3 (6)°, and similarly, the dihedral angle between the Rh1/N1*/O1* and N2*/Rh/O5* planes is 7 (6)°.

**Figure 5:**
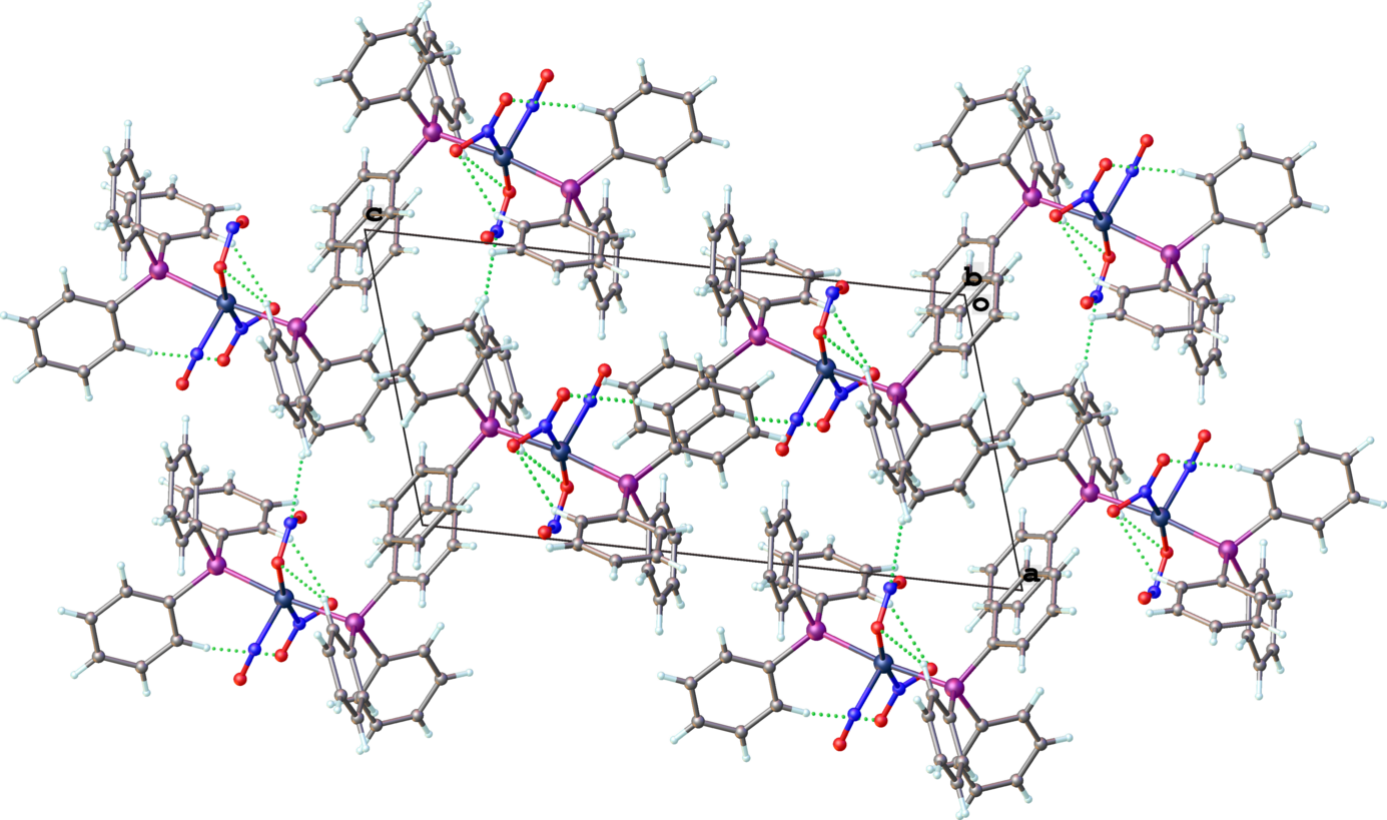
Crystal packing of the title compound viewed along the *b*-axis direction. Non-classical hydrogen-bonding inter­actions are shown as dotted green lines.

The packing diagram for the title compound is shown in Fig. 5 ▸. Both inter­molecular and intra­molecular non-classical hydrogen-bonding inter­actions are observed. All of the C—H⋯O hydrogen bonding inter­actions with the phenyl moieties and nitro and nitrito ligands are intra­molecular. Whereas all of the C—H⋯N hydrogen-bonding inter­actions between the phenyl moieties and the nitrito ligand are inter­molecular. No hydrogen-bonding inter­actions are observed with the nitrosyl ligand. All hydrogen-bonding inter­actions are summarized in Table 1 ▸ and shown as dotted green lines in Fig. 5 ▸.

## Synthesis and crystallization

The tile compound was synthesized by the previously reported procedure (Rajaseelan *et al.*, 1999 ▸). It was crystallized by slow diffusion of pentane into a CH_2_Cl_2_ solution. The complex was polymorphic with two distinct crystals recovered. Both crystals were dark green with one of them forming irregular shaped blocks and the other forming needle-like crystals. Both types of crystals had identical infrared spectra showing the presence of the nitrosyl, nitro, and nitrito ligands. The irregular shaped block crystal crystallizes in the monoclinic crystal system with large amount of disorder in the NO_*x*_ groups, and its crystal structure did not refine in a satisfactory manner. Hence, the structural set-up of the irregular shaped block crystals remains an open question. The disorder in the NO_*x*_ groups for the needle-like crystals, which solved in the triclinic *P*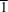 space group, was very minimal and refined well. The structure of the needle-like crystals is reported in this article.

## Refinement

Crystal data, data collection and structure refinement details are summarized in Table 2 ▸. The nitrosyl ligand exhibits positional disorder whereas the nitro and nitrito ligands show disorder across coordination sites with the disorder modelled in a 0.91:0.09 ratio. All nitro and nitrito N—O bond lengths involving disordered parts were restrained to 1.25 (2) Å. In addition, for the minor part of the nitro ligand, the O—N—O bond angle was restrained to ∼120°, with an O⋯O bond distance restrained to 2.15 (4) Å. For the disordered nitrosyl ligand, the N—O bond length in the minor part was restrained to 1.15 (2) Å. The *U*_ij_ components of all disordered atoms closer to each other than 2.0 Å were restrained to be similar, within a standard deviation of 0.002 A^2^ (Sheldrick, 2015*b* ▸).

## Supplementary Material

Crystal structure: contains datablock(s) I. DOI: 10.1107/S241431462500598X/bh4097sup1.cif

Structure factors: contains datablock(s) I. DOI: 10.1107/S241431462500598X/bh4097Isup2.hkl

CCDC reference: 2469479

Additional supporting information:  crystallographic information; 3D view; checkCIF report

## Figures and Tables

**Table 1:** Hydrogen-bond geometry (Å, °)

*D*—H⋯*A*	*D*—H	H⋯*A*	*D*⋯*A*	*D*—H⋯*A*
C2—H2⋯O5	0.95	2.27	3.200 (3)	166
C2—H2⋯O5*	0.95	2.40	3.213 (19)	144
C18—H18⋯O4	0.95	2.34	3.130 (3)	140
C21—H21⋯N2^i^	0.95	2.44	3.328 (4)	155
C32—H32⋯O2	0.95	2.40	3.118 (3)	132
C32—H32⋯O2*	0.95	2.36	3.24 (2)	154
C33—H33⋯N3*^ii^	0.95	2.38	3.21 (2)	145

Symmetry codes: (i) 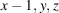; (ii) 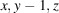.

**Table 2:** Experimental details

Crystal data	
Chemical formula	[Rh(NO)(NO_2_)_2_(C_18_H_15_P)_2_]
*M*_r_	749.48
Crystal system, space group	Triclinic, *P* 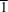
Temperature (K)	100
*a*, *b*, *c* (Å)	10.2732 (2), 10.3317 (2), 18.1514 (4)
α, β, γ (°)	90.118 (2), 105.089 (2), 118.323 (2)
*V* (Å^3^)	1619.50 (6)
*Z*	2
Radiation type	Cu *K*α
μ (mm^−1^)	5.60
Crystal size (mm)	0.17 × 0.1 × 0.03

Data collection	
Diffractometer	Rigaku XtaLAB Synergy-S
Absorption correction	Multi-scan (*SCALE3 ABSPACK*; Oxford Diffraction, 2005 ▸)
*T*_min_, *T*_max_	0.850, 1.000
No. of measured, independent and observed [*I* > 2σ(*I*)] reflections	38997, 6535, 6126
*R*_int_	0.047
(sin θ/λ)_max_ (Å^−1^)	0.625

Refinement	
*R*[*F*^2^ > 2σ(*F*^2^)], *wR*(*F*^2^), *S*	0.030, 0.082, 1.08
No. of reflections	6535
No. of parameters	487
No. of restraints	160
H-atom treatment	H-atom parameters constrained
Δρ_max_, Δρ_min_ (e Å^−3^)	0.50, −1.04

Computer programs: *CrysAlis PRO* (Rigaku OD, 2025 ▸), *SHELXT* (Sheldrick, 2015*a* ▸), *SHELXL2018/3* (Sheldrick, 2015*b* ▸), *OLEX2* (Dolomanov *et al.*, 2009 ▸) and *publCIF* (Westrip, 2010 ▸).

## full crystallographic data

### (Nitrito-κO)(nitro-κN)(nitrosyl-κN)bis(triphenylphosphane-κP)rhodium(III) Crystal data

**Table:**

[Rh(NO)(NO_2_)_2_(C_18_H_15_P)_2_]	*Z* = 2
*M**_r_* = 749.48	*F*(000) = 764
Triclinic, *P*1	*D*_x_ = 1.537 Mg m^−^^3^
*a* = 10.2732 (2) Å	Cu *K*α radiation, λ = 1.54184 Å
*b* = 10.3317 (2) Å	Cell parameters from 26769 reflections
*c* = 18.1514 (4) Å	θ = 4.9–74.4°
α = 90.118 (2)°	µ = 5.60 mm^−^^1^
β = 105.089 (2)°	*T* = 100 K
γ = 118.323 (2)°	Needle, green
*V* = 1619.50 (6) Å^3^	0.17 × 0.1 × 0.03 mm

### (Nitrito-κO)(nitro-κN)(nitrosyl-κN)bis(triphenylphosphane-κP)rhodium(III) Data collection

**Table:**

Rigaku XtaLAB Synergy-S diffractometer	6126 reflections with *I* > 2σ(*I*)
Detector resolution: 10.0000 pixels mm^-1^	*R*_int_ = 0.047
ω scans	θ_max_ = 74.5°, θ_min_ = 4.9°
Absorption correction: multi-scan (*SCALE3 ABSPACK*; Oxford Diffraction, 2005)	*h* = −12→12
*T*_min_ = 0.850, *T*_max_ = 1.000	*k* = −12→12
38997 measured reflections	*l* = −22→20
6535 independent reflections	

### (Nitrito-κO)(nitro-κN)(nitrosyl-κN)bis(triphenylphosphane-κP)rhodium(III) Refinement

**Table:**

Refinement on *F*^2^	Primary atom site location: dual
Least-squares matrix: full	Secondary atom site location: difference Fourier map
*R*[*F*^2^ > 2σ(*F*^2^)] = 0.030	Hydrogen site location: inferred from neighbouring sites
*wR*(*F*^2^) = 0.082	H-atom parameters constrained
*S* = 1.08	*w* = 1/[σ^2^(*F*_o_^2^) + (0.0475*P*)^2^ + 1.2428*P*] where *P* = (*F*_o_^2^ + 2*F*_c_^2^)/3
6535 reflections	(Δ/σ)_max_ = 0.002
487 parameters	Δρ_max_ = 0.50 e Å^−^^3^
160 restraints	Δρ_min_ = −1.04 e Å^−^^3^

### (Nitrito-κO)(nitro-κN)(nitrosyl-κN)bis(triphenylphosphane-κP)rhodium(III) Fractional atomic coordinates and isotropic or equivalent isotropic displacement parameters (Å^2^)

**Table:**

	*x*	*y*	*z*	*U*_iso_*/*U*_eq_	Occ. (<1)
Rh1	0.69455 (2)	0.72063 (2)	0.73984 (2)	0.01782 (7)	
P1	0.78304 (6)	0.84065 (6)	0.63584 (3)	0.01877 (12)	
P2	0.63593 (6)	0.61376 (6)	0.85208 (3)	0.01834 (12)	
O1	0.3977 (2)	0.5606 (3)	0.63816 (14)	0.0403 (5)	0.91
O1*	0.441 (3)	0.510 (3)	0.6490 (16)	0.037 (2)	0.09
O2	0.8146 (2)	0.6017 (3)	0.74115 (12)	0.0306 (4)	0.91
O2*	0.892 (3)	0.580 (2)	0.7594 (12)	0.0315 (18)	0.09
O3	0.9814 (4)	0.8223 (4)	0.7943 (3)	0.0385 (8)	0.91
O3*	1.002 (5)	0.808 (4)	0.800 (3)	0.036 (2)	0.09
O4	0.6922 (2)	0.94890 (19)	0.81800 (10)	0.0277 (3)	
O5	0.4959 (2)	0.8559 (2)	0.71591 (12)	0.0309 (4)	0.91
O5*	0.528 (2)	0.788 (2)	0.7215 (11)	0.0250 (12)	0.09
N1	0.5063 (4)	0.5562 (3)	0.67278 (15)	0.0296 (5)	0.91
N1*	0.566 (3)	0.533 (3)	0.6720 (15)	0.0333 (15)	0.09
N2	0.9564 (3)	0.6945 (3)	0.77560 (15)	0.0357 (5)	0.91
N2*	0.882 (3)	0.691 (3)	0.7696 (16)	0.0344 (12)	0.09
N3	0.6127 (3)	0.8576 (3)	0.75739 (13)	0.0231 (4)	0.91
N3*	0.567 (2)	0.900 (2)	0.7675 (11)	0.0261 (11)	0.09
C1	0.6388 (3)	0.8054 (3)	0.54337 (13)	0.0227 (5)	
C2	0.5094 (3)	0.8184 (3)	0.54348 (15)	0.0286 (5)	
H2	0.494092	0.836812	0.590960	0.034*	
C3	0.4032 (3)	0.8042 (3)	0.47396 (16)	0.0344 (6)	
H3	0.315916	0.813992	0.474200	0.041*	
C4	0.4236 (3)	0.7758 (3)	0.40436 (16)	0.0341 (6)	
H4	0.350085	0.765054	0.357057	0.041*	
C5	0.5524 (3)	0.7632 (3)	0.40421 (15)	0.0345 (6)	
H5	0.567554	0.744994	0.356622	0.041*	
C6	0.6591 (3)	0.7771 (3)	0.47329 (14)	0.0300 (5)	
H6	0.746170	0.767166	0.472777	0.036*	
C7	0.9129 (3)	0.7841 (3)	0.61338 (13)	0.0217 (4)	
C8	0.8534 (3)	0.6345 (3)	0.58525 (13)	0.0242 (5)	
H8	0.745487	0.566960	0.573356	0.029*	
C9	0.9521 (3)	0.5854 (3)	0.57487 (14)	0.0273 (5)	
H9	0.911444	0.484159	0.555225	0.033*	
C10	1.1108 (3)	0.6832 (3)	0.59298 (14)	0.0278 (5)	
H10	1.178146	0.648121	0.586676	0.033*	
C11	1.1700 (3)	0.8316 (3)	0.62018 (14)	0.0274 (5)	
H11	1.277986	0.898430	0.631972	0.033*	
C12	1.0721 (3)	0.8828 (3)	0.63025 (14)	0.0235 (5)	
H12	1.112988	0.984776	0.648581	0.028*	
C13	0.8930 (3)	1.0430 (3)	0.65558 (13)	0.0210 (4)	
C14	0.9389 (3)	1.1237 (3)	0.59653 (14)	0.0244 (5)	
H14	0.913317	1.073008	0.546782	0.029*	
C15	1.0216 (3)	1.2776 (3)	0.61078 (14)	0.0278 (5)	
H15	1.056194	1.332126	0.571264	0.033*	
C16	1.0544 (3)	1.3525 (3)	0.68239 (15)	0.0286 (5)	
H16	1.107521	1.458101	0.691253	0.034*	
C17	1.0093 (3)	1.2730 (3)	0.74093 (14)	0.0284 (5)	
H17	1.032255	1.324186	0.790112	0.034*	
C18	0.9313 (3)	1.1197 (3)	0.72809 (14)	0.0239 (5)	
H18	0.903358	1.065904	0.768954	0.029*	
C19	0.5170 (3)	0.6706 (3)	0.88654 (14)	0.0221 (5)	
C20	0.3787 (3)	0.6470 (3)	0.83388 (15)	0.0268 (5)	
H20	0.345315	0.594367	0.783672	0.032*	
C21	0.2902 (3)	0.6998 (3)	0.85440 (17)	0.0335 (6)	
H21	0.195776	0.682420	0.818574	0.040*	
C22	0.3393 (3)	0.7781 (3)	0.92717 (18)	0.0348 (6)	
H22	0.279499	0.816250	0.940739	0.042*	
C23	0.4751 (3)	0.8010 (3)	0.98034 (17)	0.0332 (6)	
H23	0.507462	0.853773	1.030425	0.040*	
C24	0.5645 (3)	0.7466 (3)	0.96031 (14)	0.0257 (5)	
H24	0.657231	0.761405	0.996838	0.031*	
C25	0.8054 (3)	0.6635 (2)	0.93392 (13)	0.0203 (4)	
C26	0.9306 (3)	0.8090 (3)	0.94934 (14)	0.0238 (5)	
H26	0.927203	0.879629	0.916565	0.029*	
C27	1.0593 (3)	0.8494 (3)	1.01262 (14)	0.0268 (5)	
H27	1.143967	0.948024	1.023111	0.032*	
C28	1.0653 (3)	0.7469 (3)	1.06066 (14)	0.0277 (5)	
H28	1.154064	0.775106	1.103662	0.033*	
C29	0.9420 (3)	0.6037 (3)	1.04595 (14)	0.0275 (5)	
H29	0.946168	0.533630	1.078950	0.033*	
C30	0.8113 (3)	0.5615 (3)	0.98281 (14)	0.0240 (5)	
H30	0.726371	0.463211	0.973195	0.029*	
C31	0.5316 (3)	0.4110 (3)	0.83444 (13)	0.0235 (5)	
C32	0.6032 (3)	0.3373 (3)	0.81272 (15)	0.0295 (5)	
H32	0.704272	0.393404	0.807580	0.035*	
C33	0.5282 (3)	0.1835 (3)	0.79864 (15)	0.0335 (6)	
H33	0.578905	0.134618	0.785066	0.040*	
C34	0.3792 (4)	0.1007 (3)	0.80434 (16)	0.0383 (7)	
H34	0.327126	−0.004743	0.793955	0.046*	
C35	0.3069 (3)	0.1723 (3)	0.82517 (19)	0.0418 (7)	
H35	0.204757	0.115570	0.828892	0.050*	
C36	0.3824 (3)	0.3269 (3)	0.84077 (17)	0.0344 (6)	
H36	0.332296	0.375031	0.855739	0.041*	

### (Nitrito-κO)(nitro-κN)(nitrosyl-κN)bis(triphenylphosphane-κP)rhodium(III) Atomic displacement parameters (Å^2^)

**Table:**

	*U*^11^	*U*^22^	*U*^33^	*U*^12^	*U*^13^	*U*^23^
Rh1	0.01562 (9)	0.02078 (10)	0.01968 (10)	0.01061 (7)	0.00593 (6)	0.00453 (6)
P1	0.0167 (3)	0.0223 (3)	0.0190 (3)	0.0107 (2)	0.0056 (2)	0.0042 (2)
P2	0.0156 (2)	0.0197 (3)	0.0202 (3)	0.0090 (2)	0.0054 (2)	0.0039 (2)
O1	0.0245 (10)	0.0361 (11)	0.0473 (12)	0.0111 (9)	−0.0014 (9)	−0.0047 (9)
O1*	0.027 (3)	0.032 (3)	0.041 (3)	0.010 (2)	0.000 (2)	−0.001 (3)
O2	0.0353 (9)	0.0436 (10)	0.0324 (9)	0.0301 (8)	0.0188 (8)	0.0158 (8)
O2*	0.033 (2)	0.047 (3)	0.032 (2)	0.030 (2)	0.017 (2)	0.018 (2)
O3	0.0268 (13)	0.0532 (13)	0.0279 (12)	0.0135 (10)	0.0084 (11)	0.0120 (11)
O3*	0.030 (3)	0.052 (3)	0.030 (2)	0.022 (2)	0.014 (2)	0.014 (2)
O4	0.0267 (8)	0.0257 (8)	0.0293 (8)	0.0107 (6)	0.0105 (6)	0.0010 (6)
O5	0.0298 (9)	0.0396 (10)	0.0313 (10)	0.0251 (8)	0.0056 (8)	0.0050 (8)
O5*	0.0251 (16)	0.0279 (16)	0.0267 (16)	0.0153 (15)	0.0102 (15)	0.0065 (16)
N1	0.0246 (12)	0.0271 (12)	0.0309 (12)	0.0104 (10)	0.0036 (10)	−0.0012 (9)
N1*	0.025 (2)	0.030 (2)	0.035 (2)	0.0098 (19)	0.0020 (19)	−0.0011 (19)
N2	0.0334 (10)	0.0534 (12)	0.0322 (10)	0.0274 (9)	0.0157 (9)	0.0175 (9)
N2*	0.0332 (15)	0.0499 (15)	0.0320 (15)	0.0263 (15)	0.0161 (14)	0.0159 (14)
N3	0.0231 (9)	0.0251 (9)	0.0259 (9)	0.0139 (8)	0.0105 (7)	0.0075 (7)
N3*	0.0257 (14)	0.0276 (14)	0.0276 (14)	0.0138 (13)	0.0103 (13)	0.0051 (13)
C1	0.0208 (11)	0.0220 (11)	0.0231 (11)	0.0105 (9)	0.0033 (9)	0.0044 (9)
C2	0.0213 (11)	0.0357 (13)	0.0294 (13)	0.0148 (10)	0.0067 (10)	0.0072 (10)
C3	0.0210 (12)	0.0413 (15)	0.0368 (14)	0.0143 (11)	0.0040 (10)	0.0096 (12)
C4	0.0289 (13)	0.0321 (14)	0.0281 (13)	0.0109 (11)	−0.0035 (10)	0.0059 (11)
C5	0.0428 (15)	0.0361 (14)	0.0221 (12)	0.0207 (12)	0.0033 (11)	0.0028 (10)
C6	0.0340 (13)	0.0376 (14)	0.0227 (12)	0.0218 (11)	0.0066 (10)	0.0069 (10)
C7	0.0219 (11)	0.0269 (11)	0.0204 (11)	0.0143 (9)	0.0082 (9)	0.0056 (9)
C8	0.0262 (12)	0.0279 (12)	0.0223 (11)	0.0149 (10)	0.0097 (9)	0.0070 (9)
C9	0.0335 (13)	0.0295 (12)	0.0250 (12)	0.0192 (11)	0.0106 (10)	0.0048 (10)
C10	0.0322 (13)	0.0388 (14)	0.0259 (12)	0.0252 (11)	0.0138 (10)	0.0103 (10)
C11	0.0222 (11)	0.0372 (14)	0.0271 (12)	0.0164 (10)	0.0103 (9)	0.0080 (10)
C12	0.0205 (11)	0.0266 (12)	0.0254 (12)	0.0124 (9)	0.0080 (9)	0.0060 (9)
C13	0.0162 (10)	0.0246 (11)	0.0235 (11)	0.0115 (9)	0.0048 (8)	0.0039 (9)
C14	0.0250 (11)	0.0276 (12)	0.0221 (11)	0.0145 (10)	0.0061 (9)	0.0057 (9)
C15	0.0265 (12)	0.0295 (13)	0.0256 (12)	0.0126 (10)	0.0073 (10)	0.0109 (10)
C16	0.0221 (11)	0.0250 (12)	0.0317 (13)	0.0089 (10)	0.0025 (10)	0.0056 (10)
C17	0.0250 (12)	0.0282 (12)	0.0240 (12)	0.0087 (10)	0.0040 (9)	−0.0013 (10)
C18	0.0200 (11)	0.0284 (12)	0.0214 (11)	0.0101 (9)	0.0067 (9)	0.0050 (9)
C19	0.0185 (10)	0.0210 (11)	0.0292 (12)	0.0094 (9)	0.0117 (9)	0.0080 (9)
C20	0.0214 (11)	0.0295 (12)	0.0334 (13)	0.0137 (10)	0.0120 (10)	0.0109 (10)
C21	0.0236 (12)	0.0357 (14)	0.0488 (16)	0.0173 (11)	0.0174 (11)	0.0170 (12)
C22	0.0347 (14)	0.0368 (14)	0.0514 (17)	0.0243 (12)	0.0278 (13)	0.0187 (12)
C23	0.0392 (15)	0.0328 (13)	0.0374 (14)	0.0195 (12)	0.0226 (12)	0.0080 (11)
C24	0.0251 (11)	0.0278 (12)	0.0289 (12)	0.0133 (10)	0.0146 (10)	0.0084 (10)
C25	0.0168 (10)	0.0222 (11)	0.0216 (11)	0.0091 (9)	0.0060 (8)	0.0024 (9)
C26	0.0217 (11)	0.0230 (11)	0.0281 (12)	0.0106 (9)	0.0102 (9)	0.0055 (9)
C27	0.0193 (11)	0.0245 (12)	0.0298 (13)	0.0068 (9)	0.0048 (9)	−0.0039 (9)
C28	0.0229 (11)	0.0349 (13)	0.0243 (12)	0.0161 (10)	0.0016 (9)	−0.0017 (10)
C29	0.0293 (12)	0.0299 (13)	0.0238 (12)	0.0159 (10)	0.0060 (10)	0.0055 (10)
C30	0.0229 (11)	0.0247 (11)	0.0230 (11)	0.0110 (9)	0.0060 (9)	0.0043 (9)
C31	0.0219 (11)	0.0220 (11)	0.0217 (11)	0.0092 (9)	0.0022 (9)	0.0056 (9)
C32	0.0362 (13)	0.0291 (13)	0.0257 (12)	0.0178 (11)	0.0098 (10)	0.0049 (10)
C33	0.0481 (16)	0.0293 (13)	0.0227 (12)	0.0217 (12)	0.0044 (11)	0.0034 (10)
C34	0.0445 (16)	0.0205 (12)	0.0318 (14)	0.0115 (11)	−0.0078 (12)	0.0019 (10)
C35	0.0249 (13)	0.0285 (14)	0.0553 (18)	0.0066 (11)	−0.0010 (12)	0.0124 (13)
C36	0.0215 (12)	0.0274 (13)	0.0493 (17)	0.0103 (10)	0.0061 (11)	0.0087 (11)

### (Nitrito-κO)(nitro-κN)(nitrosyl-κN)bis(triphenylphosphane-κP)rhodium(III) Geometric parameters (Å, º)

**Table:**

Rh1—P1	2.4001 (6)	C12—H12	0.9500
Rh1—P2	2.3978 (6)	C13—C14	1.400 (3)
Rh1—O2	2.1105 (19)	C13—C18	1.395 (3)
Rh1—O5*	2.09 (2)	C14—H14	0.9500
Rh1—N1	1.938 (3)	C14—C15	1.385 (4)
Rh1—N1*	1.93 (3)	C15—H15	0.9500
Rh1—N2*	2.03 (3)	C15—C16	1.387 (4)
Rh1—N3	2.019 (2)	C16—H16	0.9500
P1—C1	1.827 (2)	C16—C17	1.385 (4)
P1—C7	1.819 (2)	C17—H17	0.9500
P1—C13	1.822 (2)	C17—C18	1.379 (4)
P2—C19	1.819 (2)	C18—H18	0.9500
P2—C25	1.818 (2)	C19—C20	1.398 (3)
P2—C31	1.825 (2)	C19—C24	1.393 (3)
O1—N1	1.152 (4)	C20—H20	0.9500
O1*—N1*	1.147 (10)	C20—C21	1.381 (4)
O2—N2	1.277 (4)	C21—H21	0.9500
O2*—N2*	1.216 (18)	C21—C22	1.384 (4)
O3—N2	1.248 (5)	C22—H22	0.9500
O3*—N2*	1.23 (2)	C22—C23	1.384 (4)
O4—N3	1.251 (3)	C23—H23	0.9500
O4—N3*	1.230 (16)	C23—C24	1.399 (3)
O5—N3	1.231 (3)	C24—H24	0.9500
O5*—N3*	1.259 (18)	C25—C26	1.403 (3)
C1—C2	1.397 (3)	C25—C30	1.392 (3)
C1—C6	1.394 (3)	C26—H26	0.9500
C2—H2	0.9500	C26—C27	1.388 (3)
C2—C3	1.391 (4)	C27—H27	0.9500
C3—H3	0.9500	C27—C28	1.387 (4)
C3—C4	1.386 (4)	C28—H28	0.9500
C4—H4	0.9500	C28—C29	1.381 (4)
C4—C5	1.391 (4)	C29—H29	0.9500
C5—H5	0.9500	C29—C30	1.395 (3)
C5—C6	1.388 (4)	C30—H30	0.9500
C6—H6	0.9500	C31—C32	1.399 (4)
C7—C8	1.401 (3)	C31—C36	1.394 (4)
C7—C12	1.401 (3)	C32—H32	0.9500
C8—H8	0.9500	C32—C33	1.385 (4)
C8—C9	1.381 (3)	C33—H33	0.9500
C9—H9	0.9500	C33—C34	1.388 (4)
C9—C10	1.394 (4)	C34—H34	0.9500
C10—H10	0.9500	C34—C35	1.381 (5)
C10—C11	1.385 (4)	C35—H35	0.9500
C11—H11	0.9500	C35—C36	1.393 (4)
C11—C12	1.388 (3)	C36—H36	0.9500
			
P2—Rh1—P1	173.609 (19)	C7—C12—H12	120.0
O2—Rh1—P1	90.54 (5)	C11—C12—C7	120.0 (2)
O2—Rh1—P2	86.46 (5)	C11—C12—H12	120.0
O5*—Rh1—P1	91.4 (5)	C14—C13—P1	119.40 (18)
O5*—Rh1—P2	93.0 (5)	C18—C13—P1	121.50 (18)
N1—Rh1—P1	94.47 (8)	C18—C13—C14	119.1 (2)
N1—Rh1—P2	91.22 (8)	C13—C14—H14	120.1
N1—Rh1—O2	90.83 (10)	C15—C14—C13	119.9 (2)
N1—Rh1—N3	99.52 (10)	C15—C14—H14	120.1
N1*—Rh1—P1	90.3 (8)	C14—C15—H15	119.8
N1*—Rh1—P2	93.8 (8)	C14—C15—C16	120.4 (2)
N1*—Rh1—O5*	96.8 (9)	C16—C15—H15	119.8
N1*—Rh1—N2*	93.3 (11)	C15—C16—H16	120.1
N2*—Rh1—P1	88.1 (8)	C17—C16—C15	119.8 (2)
N2*—Rh1—P2	86.8 (8)	C17—C16—H16	120.1
N2*—Rh1—O5*	169.9 (9)	C16—C17—H17	119.9
N3—Rh1—P1	92.49 (6)	C18—C17—C16	120.2 (2)
N3—Rh1—P2	89.44 (6)	C18—C17—H17	119.9
N3—Rh1—O2	168.95 (9)	C13—C18—H18	119.7
C1—P1—Rh1	118.18 (8)	C17—C18—C13	120.5 (2)
C7—P1—Rh1	109.77 (8)	C17—C18—H18	119.7
C7—P1—C1	104.60 (11)	C20—C19—P2	117.62 (19)
C7—P1—C13	105.26 (11)	C24—C19—P2	122.60 (18)
C13—P1—Rh1	115.08 (8)	C24—C19—C20	119.6 (2)
C13—P1—C1	102.70 (10)	C19—C20—H20	119.8
C19—P2—Rh1	112.01 (8)	C21—C20—C19	120.4 (3)
C19—P2—C31	106.84 (11)	C21—C20—H20	119.8
C25—P2—Rh1	114.11 (8)	C20—C21—H21	120.0
C25—P2—C19	105.75 (11)	C20—C21—C22	120.0 (3)
C25—P2—C31	105.04 (10)	C22—C21—H21	120.0
C31—P2—Rh1	112.48 (8)	C21—C22—H22	119.8
N2—O2—Rh1	105.34 (19)	C21—C22—C23	120.4 (2)
N3*—O5*—Rh1	113.5 (16)	C23—C22—H22	119.8
O1—N1—Rh1	126.1 (2)	C22—C23—H23	120.0
O1*—N1*—Rh1	111 (2)	C22—C23—C24	120.0 (3)
O3—N2—O2	114.1 (3)	C24—C23—H23	120.0
O2*—N2*—Rh1	130 (2)	C19—C24—C23	119.6 (2)
O2*—N2*—O3*	118 (3)	C19—C24—H24	120.2
O3*—N2*—Rh1	111 (2)	C23—C24—H24	120.2
O4—N3—Rh1	113.23 (16)	C26—C25—P2	119.17 (18)
O5—N3—Rh1	126.50 (19)	C30—C25—P2	121.30 (18)
O5—N3—O4	120.3 (2)	C30—C25—C26	119.5 (2)
O4—N3*—O5*	116 (2)	C25—C26—H26	120.1
C2—C1—P1	118.11 (19)	C27—C26—C25	119.8 (2)
C6—C1—P1	122.31 (18)	C27—C26—H26	120.1
C6—C1—C2	119.4 (2)	C26—C27—H27	119.8
C1—C2—H2	120.0	C28—C27—C26	120.5 (2)
C3—C2—C1	119.9 (2)	C28—C27—H27	119.8
C3—C2—H2	120.0	C27—C28—H28	120.0
C2—C3—H3	119.7	C29—C28—C27	120.0 (2)
C4—C3—C2	120.5 (3)	C29—C28—H28	120.0
C4—C3—H3	119.7	C28—C29—H29	119.9
C3—C4—H4	120.2	C28—C29—C30	120.3 (2)
C3—C4—C5	119.6 (2)	C30—C29—H29	119.9
C5—C4—H4	120.2	C25—C30—C29	120.0 (2)
C4—C5—H5	119.8	C25—C30—H30	120.0
C6—C5—C4	120.3 (3)	C29—C30—H30	120.0
C6—C5—H5	119.8	C32—C31—P2	118.68 (19)
C1—C6—H6	119.9	C36—C31—P2	122.5 (2)
C5—C6—C1	120.2 (2)	C36—C31—C32	118.9 (2)
C5—C6—H6	119.9	C31—C32—H32	119.7
C8—C7—P1	118.47 (18)	C33—C32—C31	120.7 (3)
C8—C7—C12	119.6 (2)	C33—C32—H32	119.7
C12—C7—P1	121.65 (18)	C32—C33—H33	120.0
C7—C8—H8	120.1	C32—C33—C34	120.1 (3)
C9—C8—C7	119.7 (2)	C34—C33—H33	120.0
C9—C8—H8	120.1	C33—C34—H34	120.1
C8—C9—H9	119.7	C35—C34—C33	119.7 (3)
C8—C9—C10	120.6 (2)	C35—C34—H34	120.1
C10—C9—H9	119.7	C34—C35—H35	119.7
C9—C10—H10	120.1	C34—C35—C36	120.6 (3)
C11—C10—C9	119.9 (2)	C36—C35—H35	119.7
C11—C10—H10	120.1	C31—C36—H36	120.0
C10—C11—H11	119.9	C35—C36—C31	120.1 (3)
C10—C11—C12	120.2 (2)	C35—C36—H36	120.0
C12—C11—H11	119.9		
			
Rh1—P1—C1—C2	48.7 (2)	C9—C10—C11—C12	0.7 (4)
Rh1—P1—C1—C6	−135.99 (19)	C10—C11—C12—C7	0.4 (4)
Rh1—P1—C7—C8	64.22 (19)	C12—C7—C8—C9	0.4 (4)
Rh1—P1—C7—C12	−109.70 (19)	C13—P1—C1—C2	−79.2 (2)
Rh1—P1—C13—C14	−174.86 (16)	C13—P1—C1—C6	96.1 (2)
Rh1—P1—C13—C18	4.5 (2)	C13—P1—C7—C8	−171.37 (18)
Rh1—P2—C19—C20	54.1 (2)	C13—P1—C7—C12	14.7 (2)
Rh1—P2—C19—C24	−120.50 (19)	C13—C14—C15—C16	−2.4 (4)
Rh1—P2—C25—C26	39.3 (2)	C14—C13—C18—C17	1.9 (3)
Rh1—P2—C25—C30	−142.69 (17)	C14—C15—C16—C17	2.5 (4)
Rh1—P2—C31—C32	61.4 (2)	C15—C16—C17—C18	−0.4 (4)
Rh1—P2—C31—C36	−117.7 (2)	C16—C17—C18—C13	−1.8 (4)
Rh1—O2—N2—O3	−2.7 (4)	C18—C13—C14—C15	0.2 (3)
Rh1—O5*—N3*—O4	−4 (3)	C19—P2—C25—C26	−84.2 (2)
P1—C1—C2—C3	174.9 (2)	C19—P2—C25—C30	93.8 (2)
P1—C1—C6—C5	−174.6 (2)	C19—P2—C31—C32	−175.35 (19)
P1—C7—C8—C9	−173.66 (18)	C19—P2—C31—C36	5.6 (2)
P1—C7—C12—C11	172.88 (18)	C19—C20—C21—C22	0.7 (4)
P1—C13—C14—C15	179.58 (18)	C20—C19—C24—C23	−1.3 (4)
P1—C13—C18—C17	−177.46 (19)	C20—C21—C22—C23	−1.5 (4)
P2—C19—C20—C21	−174.10 (19)	C21—C22—C23—C24	0.8 (4)
P2—C19—C24—C23	173.17 (19)	C22—C23—C24—C19	0.6 (4)
P2—C25—C26—C27	178.60 (18)	C24—C19—C20—C21	0.6 (4)
P2—C25—C30—C29	−178.92 (18)	C25—P2—C19—C20	178.94 (18)
P2—C31—C32—C33	−179.91 (19)	C25—P2—C19—C24	4.4 (2)
P2—C31—C36—C35	178.7 (2)	C25—P2—C31—C32	−63.3 (2)
C1—P1—C7—C8	−63.5 (2)	C25—P2—C31—C36	117.6 (2)
C1—P1—C7—C12	122.6 (2)	C25—C26—C27—C28	0.1 (4)
C1—P1—C13—C14	−45.1 (2)	C26—C25—C30—C29	−0.9 (4)
C1—P1—C13—C18	134.26 (19)	C26—C27—C28—C29	−0.5 (4)
C1—C2—C3—C4	0.6 (4)	C27—C28—C29—C30	0.1 (4)
C2—C1—C6—C5	0.6 (4)	C28—C29—C30—C25	0.6 (4)
C2—C3—C4—C5	−0.7 (4)	C30—C25—C26—C27	0.6 (3)
C3—C4—C5—C6	0.8 (4)	C31—P2—C19—C20	−69.5 (2)
C4—C5—C6—C1	−0.7 (4)	C31—P2—C19—C24	115.9 (2)
C6—C1—C2—C3	−0.6 (4)	C31—P2—C25—C26	162.96 (19)
C7—P1—C1—C2	171.12 (19)	C31—P2—C25—C30	−19.0 (2)
C7—P1—C1—C6	−13.6 (2)	C31—C32—C33—C34	1.4 (4)
C7—P1—C13—C14	64.2 (2)	C32—C31—C36—C35	−0.4 (4)
C7—P1—C13—C18	−116.51 (19)	C32—C33—C34—C35	−0.9 (4)
C7—C8—C9—C10	0.7 (4)	C33—C34—C35—C36	−0.2 (4)
C8—C7—C12—C11	−1.0 (4)	C34—C35—C36—C31	0.9 (4)
C8—C9—C10—C11	−1.3 (4)	C36—C31—C32—C33	−0.8 (4)

### (Nitrito-κO)(nitro-κN)(nitrosyl-κN)bis(triphenylphosphane-κP)rhodium(III) Hydrogen-bond geometry (Å, º)

**Table:**

*D*—H···*A*	*D*—H	H···*A*	*D*···*A*	*D*—H···*A*
C2—H2···O5	0.95	2.27	3.200 (3)	166
C2—H2···O5*	0.95	2.40	3.213 (19)	144
C18—H18···O4	0.95	2.34	3.130 (3)	140
C21—H21···N2^i^	0.95	2.44	3.328 (4)	155
C32—H32···O2	0.95	2.40	3.118 (3)	132
C32—H32···O2*	0.95	2.36	3.24 (2)	154
C33—H33···N3*^ii^	0.95	2.38	3.21 (2)	145

Symmetry codes: (i) *x*−1, *y*, *z*; (ii) *x*, *y*−1, *z*.
